# The Potential for Trypsin Inhibitor Expression in Leaves to Convey Herbivory Deterrence in Soybean

**DOI:** 10.3390/plants14040617

**Published:** 2025-02-18

**Authors:** Audrey E. Birdwell, Sebe A. Brown, Gino J. D’Angelo, Mitra Mazarei, Charles Neal Stewart

**Affiliations:** 1 Department of Plant Sciences, University of Tennessee, Knoxville, TN 37996, USA; abirdwe4@vols.utk.edu (A.E.B.); mmazarei@utk.edu (M.M.); 2 Department of Entomology and Plant Pathology, University of Tennessee, Jackson, TN 38301, USA; sbrow175@utk.edu; 3 Warnell School of Forestry and Natural Resources, University of Georgia, Athens, GA 30602, USA; gdangelo@uga.edu; 4 Center for Agricultural Synthetic Biology, University of Tennessee, Knoxville, TN 37996, USA

**Keywords:** *Glycine max*, proteinase inhibitors, overexpression, herbivorous pests, insect, deer, field trials

## Abstract

Soybean (*Glycine max*) is the most widely grown legume crop in the world, providing important economic value. Pest herbivory damage by insects and mammalian wildlife, in particular the white-tailed deer (*Odocoileus virginianus*), limits yields in soybean. Incorporating trypsin inhibitors (TIs) as plant protectant against herbivory pests has been of interest. We previously showed that the overexpression of soybean TIs in soybean conferred insect deterrence under greenhouse experiments. In this study, we examined the potential of transgenic TI-overexpressing lines in deterring insects under field conditions at Knoxville, Tennessee. Our results indicate that the overexpression of TI could lead to a significant reduction in leaf defoliation of the transgenic compared to non-transgenic lines without negatively impacting plant growth and yield under field conditions. Furthermore, we extended our study by comprehensive evaluation of these transgenic plants against the white-tailed deer herbivory in a separate field setting at Jackson, Tennessee, and with controlled deer feeding experiments. No significant differences in growth characteristics were found between transgenic and non-transgenic lines under field conditions. There were also no significant differences in deer deterrence between transgenic and non-transgenic lines in ambient deer herbivory field or controlled deer feeding trials. Our study provides further insights into more exploration of the role of TI genes in pest control in this economically important crop.

## 1. Introduction

Soybean (*Glycine max*) is one of the most economically important crops worldwide. Herbivorous pests are damaging and limit yields in soybean. Several insect pests are known as the major defoliator for soybean yield losses [1]. The other important herbivorous pest is white-tailed deer (*Odocoileus virginianus*), causing extensive damage in terms of yield losses in soybean in the Americas [2,3].

Effective strategies exist to manage damage from insect pests, but these options may not be sustainable and can result in harm to both humans and the environment. Yet, effective management strategies against the white-tailed deer, an anomalous yet destructive pest to soybean in the Midwest, Midsouth, Northeast, and Southeast of the United States, do not exist. Soybean farmers go to great lengths to protect their crops from herbivores, but pest control can be expensive, labor intensive, ineffective, dangerous, or lead to unintended consequences [4,5,6]. Soybean yield loss due to deer damage is also costly, with one model based on indemnity payments estimating that wildlife damage in the United States caused approximately USD 323.9 million in 2018 alone [2]. Another study examining wildlife damage indemnity payments in soybean for the past two decades indicated that this problem is increasing, likely because of the loss of habitat and food sources [2,3]. In 2011, wildlife damage indemnity payments for soybean crops in Missouri, Kentucky, Arkansas, Tennessee, Mississippi, Alabama, and Georgia was USD 1 million. In 2022, payments had increased to USD 4.8 million. The wildlife damage reported in these indemnity payments is almost entirely from white-tailed deer, with some damage also resulting from wild hogs [3].

Furthermore, higher rates of deer defoliation have been shown to correlate with increased amounts of herbivorous insects, as well as allowing light to reach the soil, which increases weed pressure, in turn negatively impacting yield [2,7]. The significance of this yield loss is a two-fold economic burden on soybean farmers, as both herbivore damage and strategies to address damage are multimillion-dollar issues.

While foliar insecticides can be effective in reducing insect herbivory, they can also affect the environment in unintended ways, such as leaching into water and soil, as well as causing harm to non-target organisms [4]. An incorporated plant protectant could mitigate these off-target effects caused by insecticides. Soybean that has been genetically engineered to express *Bacillus thuringiensis* (Bt) toxins [8] could offer more direct protection, but due to its highly specific mode of action, there is a greater risk for insects to evolve a resistance [9].

Strategies to mitigate deer damage also have several downsides, foremost being that they are generally ineffective. Common methods of control include depredation hunting, fencing, herbivory deterrent compounds (e.g., blood, urine, putrid egg solids), and optical deterrents [10,11,12,13]. Depredation hunting is often qualitative and does not ensure that populations are reduced below the biological carrying capacity. This leads to unstable fluctuations in deer numbers, which puts further strain on crops as a food source [14]. Herbivory-deterrent compound repellents may be considered hazardous or unsanitary to humans and require reapplication as they break down in the environment. Optical deterrents are also still used in many farms [12], but deer deterrent technology may finally progress to the level of other modern pest-deterrence strategies in other major crops.

Because deer are adaptable and intelligent animals, each of these solutions is only effective in the short term, if at all. Deer feed by preference, consuming all the most nutritious food available before moving on to their second choice. Their natural food sources consist mostly of browsing young twigs, leaves, and forbs that grow at the edge of forested areas [14,15]. In the absence of an ecosystem that can provide enough of this preferred forage, deer readily will consume row crops which are accessible and meet their energy requirements, such as wheat or soybean [16]. If a source of forage has antinutritional or toxic aspects, deer will strive to keep their intake of that forage below the threshold of toxicity [17]. Deer require a diet high in easily digestible protein [18], which also makes crop plants an apt feed. In many agroecosystems, soybean, wheat, or other crops are the optimal source of nutrients, and can compose over three-fourths of deer’s diets [16]. Deer are highly selective in the balance of their diets, carefully maximizing nutrients and minimizing toxins. Deer optimize their feeding efficiency by remembering and continuously seeking nutritionally preferable forage in the environment. Consequently, deer also identify nutritionally poor and unsuitable forage, and avoid their consumption, as well as the areas where they are located [18].

One promising candidate incorporating a broad range of plant protectants for soybean are plant proteinase inhibitors. Proteinase inhibitors prevent the digestion of protein and deplete the supply of digestive enzymes in an animal’s gut [19]. Soybean, along with many other plant species, naturally contain proteinase inhibitors as an herbivory defense mechanism but are mainly concentrated in reproductive tissues, e.g., seeds and tubers [20,21,22]. One important class of proteinase inhibitors are trypsin inhibitors (TIs). TIs have been thoroughly studied as an incorporated crop protectant against insects, and their effects are largely anti-nutritional instead of abiotic, although they significantly reduce pest damage to transgenic host plants [23,24,25].

We previously investigated the functional role of soybean TIs in plant defense against insects by the overexpression of several individual TI genes in soybean using detached leaf-punch feeding bioassay and whole soybean plant feeding bioassay under greenhouse conditions. We showed a significantly reduced leaf defoliation in the transgenic compared to non-transgenic plants [26]. However, experiments conducted under such conditions cannot predict how the plants might perform in the field. Assessing agronomic performance in the field is a necessary validation step for genetically engineered plants as greenhouse observations are not always predictive of field performance.

For that, in the present study, four promising TI-overexpressing transgenic lines from our greenhouse study [26] were selected for further analysis under the field conditions in Knoxville, Tennessee. We further examined the potential of these TI-overexpressing lines against the white-tailed deer in a separate field experiment at Jackson, Tennessee. We also performed controlled feeding assays with tame deer to more directly study behavior and quantify animal feeding preferences.

## 2. Results

### 2.1. Ambient Insect Herbivory Field Trial

#### 2.1.1. Growth Characterization

The mean of seed weight and plant height of 48 plants from the eight replicate plots per line and dry aboveground biomass of two plants from each plot (a total of 16 plants per line) was determined. ANOVA statistical analysis showed that there were no significant differences for seed weight (*p* = 0.4806, *F* = 0.84, df = 39), plant height (*p* = 0.4493, *F* = 0.90, df = 39), and dry aboveground biomass (*p* = 0.2642, *F* = 1.38, df = 39) among the transgenic and wild-type lines (Table 1).

#### 2.1.2. Insect Herbivory

The herbivorous insect species observed in the four conducted population surveys were grouped by damage type. Pod feeders and piercing-sucking insects were observed in adult form and defoliators were observed in larval form. Pod feeders included stink bug (*Hemiptera: Pentatomidae*) and bean leaf beetle (*Cerotoma trifurcate*). Piercing–sucking included scale (*Coccoideas* spp.), leaf hoppers (*Cicadellidae* spp.), and kudzu bugs (*Megacopta cribraria*). Defoliators included corn earworm (*Helicoverpa zea)* and cabbage looper (*Trichoplusia ni*). Aphids (*Aphis* spp.) were also observed consistently across all lines. The herbivorous insects with more than ten observations over the course of the population surveys were recorded (Table 2). The collection of the data was based on observations, and as such, is descriptive.

**Figure 1:**
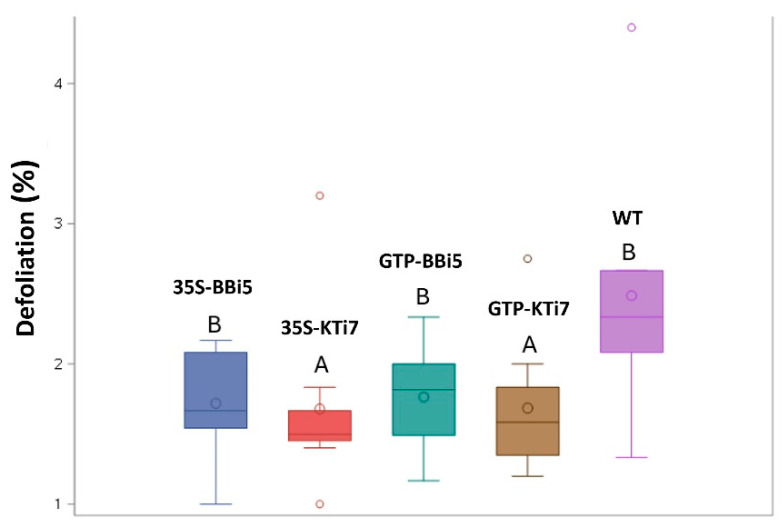
Boxplot showing the distribution of average test scores for the defoliation of transgenic trypsin inhibitor (TI)-overexpressing (35S-KTi7, GTP-KTi7, 35S-BBi5, and GTP-BBi5) and wild-type (WT) lines across three time points (13 July, 1 August, and 15 August 2023) in ambient insect herbivory soybean field trial. Boxes represented by different letters are significantly different at *p* ≤ 0.05 as tested by ANOVA with SAS software.

Based on ANOVA statistical analysis, the defoliation of the transgenic 35S-KTi7 and GTP-KTi7 lines were significantly less compared to wild type, with *p* values of 0.0236 and 0.0343, respectively. The other two transgenic (35S-BBi5 and GTP-BBi5) lines were insignificantly different from the wild type by tenths or hundredths of a decimal place: 35S-BBi5 (*p* = 0.0544) and GTP-BBi5 (*p* = 0.0891, *F* = 2.88, df = 28; Figure 1).

### 2.2. Ambient Deer Herbivory Field Trial

#### 2.2.1. Game Camera Footage

Most of the videos captured the motion of birds in the field, as well as the motion caused by weather and farm operations in the background. Out of the ~1500 videos captured, only twenty videos featured deer, and only four videos featured deer consuming soybean. Twenty-four days after planting, a group of deer was observed browsing the center of the soybean field at night. This instance was captured by both cameras. Another video of a single deer browsing the center of the field was captured two months and twenty days after planting. This instance was captured by both cameras. The other sixteen videos show deer walking through the field, moving around the edges of the field, or browsing on other adjacent plant material. For example, twenty days after planting, when the soybean were in the V3 growth phase, two videos captured a group of deer grazing on the grass in the fallow region surrounding the field in the midmorning. Another video two months later showed deer browsing in the same location.

#### 2.2.2. Yield

The total weight of seeds harvested from each plot (containing 960 plants) was determined. The seed yields were low due to the late planting date and the lack of rainfall during the growing season. Based on ANOVA statistical analysis, there were no significant differences between the transgenic and wild-type lines (*p* = 0.6437, *F* = 0.63, df = 39; Table 3).

#### 2.2.3. Defoliation

Data from the two timepoints June 28, 2024, and July 8, 2024, were used for ANOVA statistical analysis. None of the transgenic lines exhibited a significant effect on the percent of plants that were defoliated by deer compared to wild type (*p* = 0.5928, *F* = 0.71, df = 79; Table 4).

### 2.3. Controlled Deer Feeding Trials

Total reduction in consumed transgenic and wild-type leaf materials was summed from the 30 min interval measurements and recorded for each deer each day of the feeding trials. Based on ANOVA statistical analysis, there were no significant differences in the feeding preference between transgenic and wild-type lines for either the first (*p* = 0.6025, *F* = 1.16, df = 95) or second trial (*p* = 0.7582, *F* = 0.76, df = 95). There was also high variability in feeding preference among the different days of the trials (Table 5).

## 3. Discussion

Soybean is an important crop in agricultural production where pest damage limits yields in soybean. There has been interest in incorporating trypsin inhibitors (TIs) as the plant protectant against herbivory pest [23,24,25]. In the present study, we evaluated the field performance of four TI-overexpressing soybean lines selected from our previous greenhouse study [26] against insects and white-tailed deer.

### 3.1. Ambient Insect Herbivory Field Trial

Field experiments are especially important for modified plants to ensure that the agronomic performance is not compromised by their genetic modifications. Our field study revealed consistencies in growth phenotypes between greenhouse [26] and field-grown transgenic lines. These results are congruent with our previous study, where the growth parameters of these lines were determined under greenhouse conditions, suggesting there is no significant environmental influence on the growth of TI-overexpressing transgenic soybean lines. Furthermore, the herbivorous insect species observed in our population surveys were consistent with the common insects that cause soybean yield loss [27,28,29,30,31].

Two of the transgenic lines (35S-KTi7 and GTP-KTi7) showed a significant reduction in defoliation compared to wild-type soybean. Yet, the overall defoliation observed at this field site was low and below the economic threshold for yield loss [32]. Distinctly low defoliation in both transgenic and wild-type lines may indicate that there may have been other ecological interactions responsible for reducing defoliation throughout the entire field for the following reasons. The large fallow region required in the USDA APHIS BRS environmental release permit may have also hindered insect movement into the field, as the total areas of the fallow zone was wider than the field dimensions on all sides. The fallow region was also kept free of any plants or ground cover and mainly consisted of bare soil. However, the insect population surveys confirmed the consistent presence of common soybean insect pests throughout the growing season, suggesting that there may be an ecological explanation for the low defoliation observed across all lines. This notion is supported by the consistent presence of notable predatory insect species. A population of four to five gray treefrogs (*Hyla chrysoscelis*) were observed in each of the four insect population surveys. Other predatory species such as assassin bugs (*Reduviidae* spp.), ladybugs, (*Coccinellidae* spp.), and spiders including jumping spiders (*Salticidae* spp.), crab spiders (*Thomisidae* spp.), and lynx spiders (*Oxyopidae* spp.) were also observed in multiple insect population surveys. The consistent presence of predatory species may partially explain the low levels of defoliation across transgenic and wild-type lines. Foliar pesticides increase the mortality of both pest and predator species. Moreover, after predatory species are lost in an agroecosystem, fecund pest populations can rapidly repopulate [33]. The diversity of predatory species provides the compounded biocontrol of insect pests, which can result in yield protection [34]. The overexpression of TI genes in soybean has shown a significant reduction in lepidopteran larval weight and delay development in insect feeding bioassays but does not usually lead to insect mortality [24,25,26,35]. Crops containing antinutrients that hinder digestion and impede the development of herbivorous insects, leaving them in lifecycle stages that are vulnerable to predators for longer periods of time, potentially increasing their risk of becoming prey [36,37,38]. Transgenic TI-overexpressing soybean could reduce insect herbivory in field conditions, not only by deterring feeding and weakening pest insects, but also by retaining conditions that support predatory species. This potential multi-level interaction would need to be examined in further research. Transgenic TI-overexpressing soybean may provide protection from insect herbivores in sensitive ecosystems. Unlike foliar insecticides that can result in harm to off-target organisms [4], transgenic TI-overexpressing soybean would only affect insects that are actively engaging in herbivory. These transgenic soybean would be well suited for fields located near aquatic ecosystems such as wetlands, rivers, and lakes [39]. In this setting, transgenic TI-overexpressing soybean could significantly reduce defoliation by insect pests without harmful off-target effects to aquatic ecosystems associated with the application of foliar insecticide sprays [4,33].

### 3.2. Ambient Deer Herbivory Field Trial

Deer feeding behavior detected in the field was consistent with the literature. The majority of herbivory was observed within the first two weeks after plant emergence, and leveled off as the plants matured, as established by deCalesta [15]. Deer readily and repeatedly consumed transgenic TI-overexpressing lines. One explanation for this continued consumption is the category of deterrent that TIs exert on herbivores. TIs are considered a conditioned-aversion repellent, which causes feelings of illness, digestive discomfort, and post-consumption malaise [13]. Avoidance behavior is learned by association of sickness or malaise after consumption of the plants, which may take multiple instances of feeding [13]. This explanation would be congruent with the two instances of early-season herbivory by a group of females (does) followed by a lack of deer activity recorded on the game cameras. This behavior may represent deer learning the effects of the transgenic soybean and avoiding it for the rest of the growing season. However, because the deer did not differentiate between plots, this potential avoidance behavior is not reflected in the data. The deer likely did not experience the deterrent effects of the TI overexpression quickly enough to potentially learn the difference between transgenic and wild-type lines.

Also, the location of the plots in the field was more distinctly related to the percentage of plants defoliated by deer. Blocks one and eight were located at the outer edges of the field, where deer damage is known to be highest [15]. The only notable difference in deer feeding behavior on soybean in this field site was the lack of stand loss. Stand loss occurs when deer defoliate the entire shoot portion of a young soybean plant, leaving only a short section of hypocotyl. This form of herbivory is fatal to individual soybean plants, and is the main cause of yield loss from deer damage [15]. Young soybean plants can partially recover from defoliation above the cotyledonary nodes, which usually results in stunted growth, delayed maturity timing, and lower yield [40]. Soybean stand loss from deer damage has been consistently observed at this field site in recent years, but did not occur during this experiment.

Furthermore, perhaps a different field design is needed for studying the potential of TIs as a deer deterrent. Randomized block designs are a standard method for examining insect herbivory on multiple lines of transgenic crops compared to nontransgenic crops in field conditions [41,42,43]. Randomized block designs have also been used in field trials to quantify deer herbivory and preference between forage options [13,17,44]. However, because of the high similarity of the transgenic lines, delayed mode of action for deterrence, and overall low percentage of nontransgenic soybean in the field trial (one-fifth of total plants), deer may not have been able to differentiate between the effects of transgenic and nontransgenic soybean in a randomized block design.

### 3.3. Controlled Deer Feeding Trials

Out of the five-day trials, on average, deer consumed more wild-type than transgenic soybean leaf material. However, on day four, the average transgenic soybean consumed was nearly quadruple that of the wild type. No abnormal conditions or behaviors were observed on day four of the feeding trial that would explain this reversal in trend. A second feeding trial was performed to ascertain whether the behavior observed in the first feeding trial reflected a pattern that was disturbed by an unknown factor. The second feeding trial ascertained that the feeding behavior observed in the first trial was representative and would likely be observed in any further repetitions of this experiment. During the second feeding trial, the deer consumed more wild-type than transgenic soybean on three of the five days, and more transgenic soybean on two of the five days. The total amount of each option consumed during each day was more consistent during the second trial compared to the first trial. No clear preference between transgenic and wild type soybean could be discerned from the total amount of each option consumed per day in the feeding trials. Deer readily and repeatedly consumed transgenic soybean leaf materials in both feeding trials. Perhaps the simple two-option controlled feeding study could not be well adjusted to represent the deer damage in an agronomic field setting.

Moreover, live plants may have better reflected deer feeding behavior to TI-overexpressing soybean opposed to frozen and thawed leaves (as required to devitalize transgenic plants). A longer trial period in an environment that the deer are more accustomed to may also yield more accurate behavioral results.

Yet, despite the lack of significant impacts, the controlled deer feeding assays may have potential to be used as prescreening for plant protectants before developing transgenic plants. In these assays, wild-type soybean treated with a potential deterrent could be examined for the deterrent’s efficacy before attempting the genetic transformation of plants. These assays could also be used to determine the necessary concentration of the deterrent or examine the additive effects of multiple deterrent compounds acting together. Controlled deer feeding studies also allow the close monitoring of animals to assess behavioral changes in response to deterrents.

In conclusion, we showed that the overexpression of TI could lead to improved insect resistance in soybean without negatively impacting plant growth and yield under field conditions. We further examined the potential against deer herbivory in a field setting as well as under controlled feeding experiments. Despite the lack of significant impacts on deer herbivory, our results could represent useful progress towards the development of transgenic crops with incorporated protectants for deer deterrence. Further research on TIs enhance our understanding of the factors associated with reducing pest herbivory damage.

## 4. Materials and Methods

### 4.1. Plants

Homozygous T5 and T6 seeds from four promising TI-overexpressing transgenic lines [26] were used in field experiments. Included were two lines with TI expression under the control of CaMV 35S promoter (35S-KTi7 and 35S-BBi5) and two lines under the control of soybean RBCS-SRS4 green tissue-specific promoter (GTP-KTi7 and GTP-BBi5) (Table 6) with high levels of transgene expression in leaves (up to 18-fold increase in expression compared to non-transgenic wild type) [26]. Furthermore, these transgenic lines demonstrated high levels of TI enzyme activity in leaves: 35S-KTi7 (43-fold increase in enzyme activity); 35S-BBi5 (37-fold increase); GTP-KTi7 (38-fold increase); and GTP-BBi5 (28-fold increase) compared to the non-transgenic wild type [26]. The wild-type Tennessee-adapted soybean line ‘TN15-5007’ [45] used for the production of transgenic lines [26] was used as control. For controlled deer-feeding studies, the homozygous T6 seeds of the transgenic line with KTi7 expression under the control of CaMV 35S promoter (Table 6) with the highest level of transgene expression [26] was used.

### 4.2. Characterization in Ambient Insect Field Conditions

#### 4.2.1. Field Design

**Figure 2:**
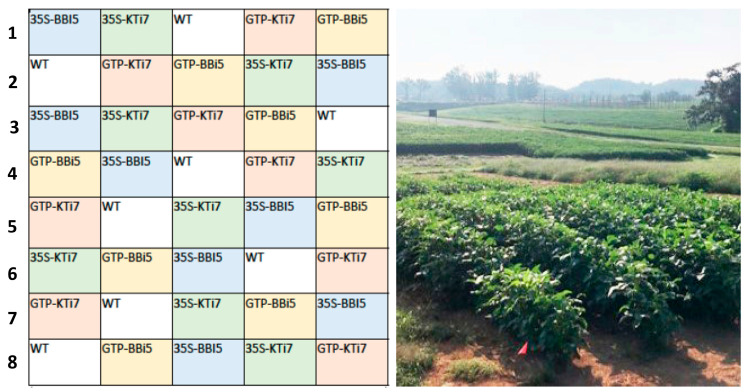
Field design of trypsin inhibitor (TI)-overexpressing (35S-KTi7, GTP-KTi7, 35S-BBi5, GTP-BBi5) and wild type (WT) lines in ambient insect herbivory soybean field trial. The field was planted on 6 June 2023, at the East Tennessee Research and Education Center (ETREC) in Knoxville, Tennessee (35.903094, −83.959253). Transgenic and WT lines were arranged in a randomized block design. Eight replicate plots were included for each transgenic and WT lines. Each individual plot measured 76.3 cm by 137 cm, and contained six plants in one row in middle of the plot with the plant spacing of 22.86 cm. The field was bordered by a fifteen-foot fallow region as required in the USDA APHIS BRS environmental release permit.

The field experiment was conducted at the East Tennessee Research and Education Center (ETREC) Knoxville, Tennessee (35.903094, −83.959253). Homozygous T5 seeds of transgenic and wild-type lines were planted in a randomized block design in eight replicate blocks. The field contained a total of 40 plots (Figure 2). Each individual plot measured 76.3 cm by 137 cm, and contained six plants in one row in the middle of the plot with the plant spacing of 22.86 cm. Seeds were planted by hand on 6 June 2023. The field was bordered by a fifteen-foot fallow region, as required in the USDA APHIS BRS environmental release permit.

No irrigation, insecticide, herbicide, or fertilizer was used at any time during the growing season and weed control was performed using hand tools. Two weeks after planting, any seeds that failed to germinate were replaced with new seeds. The plants were regularly monitored and allowed to reach maturity. The plants were individually harvested by hand on 4–9 October 2023.

#### 4.2.2. Insect Herbivory Quantification

To confirm and quantify insect pest damage in the field, two approaches were used. Defoliation ratings were used to score insect damage to plants, and insect population surveys were used to confirm the presence of common soybean pests. Defoliation ratings were taken on three time points: 13 July 2023, 1 August 2023, and 15 August 2023. Each plant was visually scored by selecting a representative leaf from the top, middle, and bottom of the plant. The most and least defoliated leaves were not considered, and the remaining leaf was used for rating. The defoliation rating was determined using a reference sheet with the leaf area removed quantified as a percentage. The reference sheet was generated with ImageJ2 and specific to soybean [46]. The mean of the defoliation measurements for each line across the three measurement time points was determined. Data were analyzed using an ANOVA and log transformed to meet normalcy assumptions with a Dunnett post hoc test. Population surveys were performed on four time points: 7 July 2023, 8 August 2023, 22 August 2023, and 5 September 2023. The top and bottom of each leaf, petiole, and the stem were carefully observed on each plant, and the herbivorous insects were recorded.

#### 4.2.3. Growth Characterization

The plants were allowed to reach full maturity in the field, and then the plant height was measured for each individual plant on 28 September 2023. Once the plants were fully dried in the field, individual plants were either stripped of pods or cut at the base and placed into a pre-labeled bag. The harvested material was taken back to the laboratory and processed by hand. Seed weight and aboveground biomass were determined.

### 4.3. Ambient Deer Herbivory Field Trial Design and Planting

#### 4.3.1. Field Design

**Figure 3:**
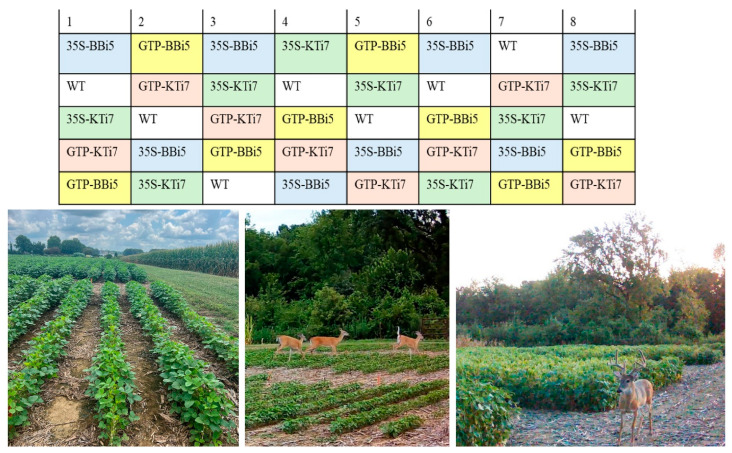
Field design of trypsin inhibitor (TI)-overexpressing (35S-KTi7, GTP-KTi7, 35S-BBi5, and GTP-BBi5) and wild-type (WT) lines in ambient deer herbivory soybean field trial. The field was planted on 14 June 2024, at the West Tennessee Research and Education Center (WTREC) in Jackson, Tennessee (35.622250, −88.850200). Transgenic and WT lines were arranged in a randomized block design. Eight replicate plots were included for each transgenic and WT lines. Each individual plot measured 3.05 m by 9.14 m, included four rows of 240 seeds, planted at a density of 24 seeds per meter, and contained 960 plants. The field was bordered by a fifteen-foot fallow region as required in the USDA APHIS BRS environmental release permit.

The field experiment was conducted at the West Tennessee Research and Education Center (WTREC) Jackson, Tennessee (35.622250, −88.850200) with a history of consistent and destructive deer herbivory to soybean during prior growing seasons. Deer had been observed to enter the field from the edge of the forest and browse plants throughout the entire field. The field trial was performed on an agronomic scale, with a planting density comparable to commercial farming. Homozygous T6 seeds of the transgenic and wild-type lines (Table 6) were planted on 14 June 2024, in a randomized block design in eight replicate blocks. The field contained a total of 40 plots (Figure 3). Each individual plot measured 3.05 m by 9.14 m, and contained four rows of 240 seeds, planted at a density of twenty-four seeds per meter. Each plot contained a total of 960 plants.

The field was oriented with block one aligned to face the forest, block eight facing an open field, and the other two sides of the field facing cotton and corn (Figure 3). The field was bordered by a fifteen-foot fallow region as required in the USDA APHIS BRS environmental release permit. The field was treated with pre-emergent, herbicide, and plots were marked with stakes after emergence. All non-target pests were controlled following the University of Tennessee’s recommendations. Two game cameras (Browning Spec Ops Elite HP5; Browning Trail Camera; Arnold, MI, USA) were placed on the outer edges of the field and set to capture ten-second videos when motion was detected for the duration of the experiment to observe the deer feeding behavior. Plants were allowed to reach full maturity in the field and then each plot was harvested mechanically. The total weight of seeds from each plot was determined.

#### 4.3.2. Data Collection

**Figure 4:**
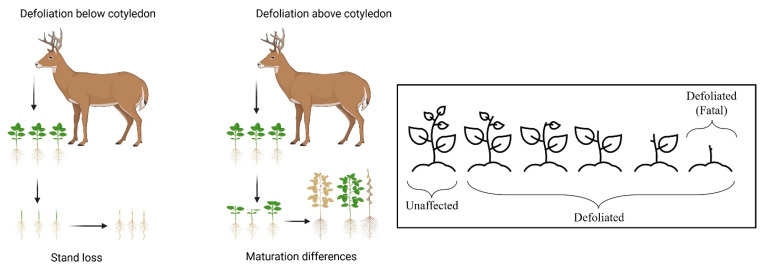
Schematic diagram of damage phenotypes by deer defoliation in ambient deer herbivory soybean field trial.

Deer defoliation was measured twice during the growing season: on 28 June 2024, two weeks after planting, and on 8 July 2024, approximately three weeks after planting. Deer predation was measured by counting the number of emerged soybean plants that were defoliated per six-meter row in each plot. Deer feeding in the early stages of soybean growth is visually distinct (Figure 4) and can be easily identified. This method of quantifying deer defoliation to soybean is frequently used in other studies [7,44,47]. The data were collected by placing a three-meter measuring tape between the two middle rows of each plot and counting the number of defoliated soybean plants in both adjacent rows. The percentage of defoliated soybeans out of the total emerged was used for statistical analysis.

#### 4.3.3. Game Cameras

From the planting to the end of the R6 (the beginning of the full seed growth stage), the two motion-activated game cameras captured twenty ten-second videos of deer activity in and around the field out of 1500 total videos captured. The game cameras were able to capture motion at a range that encompassed the entire length of the field.

### 4.4. Controlled Deer Feeding Trials

#### 4.4.1. Location

The experiments were conducted in collaboration with the University of Georgia’s Whitehall Deer Research Facility at Athens, Georgia. Cafeteria-style trials were carried out with tame deer that were presented a choice between transgenic (35S-KTi7) and wild-type lines. Cafeteria-style trials involve presenting animals with free access to a variety of food options in order to observe and quantify preference. A total of two feeding trials were performed following the same methods on 1–5 July and 30 September–4 October 2024.

#### 4.4.2. Production of Plant Materials

Homozygous T6 seeds of the transgenic (35S-KTi7) and wild-type lines were grown in the greenhouse and harvested at the VN phase. Once the sufficient plant mass was established (33 kg wild type and 27 kg transgenic soybean), the soybean plants were cut at the base and packed tightly into large sealable freezer bags, then flash frozen and devitalized using liquid nitrogen on 17 June and 29 August 2024. The freezer bags were then packed into Styrofoam coolers with dry ice and shipped overnight to the facility for the feeding trials. The frozen leaves were immediately stored in a −20 °C freezer upon arrival.

#### 4.4.3. Feeding Trial Design

A doubly repeated randomized block design was used. A total of ten deer participated in each feeding trial. The trials were conducted between 7:00 a.m. and 12:00 p.m., and hay/regular feed was removed between approximately 6:00 and 8:00 p.m. the night before each trial, allowing for twelve hours of fasting between each trial. The fasting period was included to encourage involvement and curiosity toward the soybean leaves. Hay/normal feed was replaced after each trial.

#### 4.4.4. Acclimatization Phase

First, twelve tame deer participated in a three-day acclimation trial to select suitable subjects and familiarize the deer with the experimental procedure on 26–28 June and 25–27 September 2024. During this acclimation trial, the deer were presented with one pan containing 100 g of wild-type soybean for 90 min. At 30 min intervals, the pan was weighed, the amount of soybean consumed was recorded, and the pan was refilled to 100 g. After this three-day acclimation trial, the deer were given a two-day rest period in compliance with the animal use permit conditions. From the data collected in the acclimation trials, the ten tame deer that were the most amenable to experimental procedures were selected out of the original twelve for the choice trials. The deer that participated in the trials included nine females and one male, all one and a half years of age or older. After the two days of rest, the choice trials began.

#### 4.4.5. Trial Implementation

**Figure 5:**
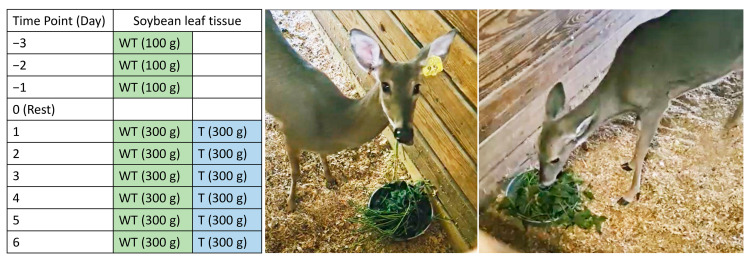
Experimental design for cafeteria-style deer feeding trials with tame deer presented a choice between trypsin inhibitor (TI)-overexpressing (35S-KTi7) and wild-type (WT) soybean leaf tissue.

The choice trials followed the same feed and fasting schedule as the acclimation trials and took place over five consecutive days. Each day, ten deer were presented with two pans of soybean: one pan containing 300 g of transgenic soybean and one pan containing 300 g of wild-type soybean for a 90 min period. At 30 min intervals, the pans were weighed, the amount consumed from each pan was recorded, and each pan was refilled to 300 g. The pans were refilled at 30 min intervals to ensure there would be a remainder for statistical analysis. Total reduction in soybean from each pan over the 90 min period was measured for each of the ten deer for the five consecutive days of the trials (Figure 5). The location of the pans in the stall was randomized each day to avoid any placement bias. Water loss from the plant material during the 30 min intervals was observed to be negligible in the raw data, as the weight of the leaves did not change from the beginning to the end of the interval if the deer not consume any soybean. During the second trial, the last of the three reduction measurements during the 90 min period were not recorded for deer W198 and Bambam on day four. The sum of the first two reduction measurements fell within the normal variation of the data, and thus was used for statistical analysis.

### 4.5. Statistical Analysis

Means were analyzed in SAS version 9.4 (SAS Institute Inc., Cary, NC, USA) using an analysis of variance (ANOVA) and log transformed to meet normalcy assumptions, with a Dunnett post hoc test. Differences were considered statistically significant at *p* ≤ 0.05.

## Figures and Tables

**Table 1:** Growth characteristics of transgenic trypsin inhibitor (TI)-overexpressing (35S-BBi5, 35S-KTi7, GTP-BBi5, GTP-KTi7) and wild type in ambient insect herbivory soybean field trial.

Line	Seed Weight (g)	Plant Height (cm)	Aboveground Biomass Weight (g)
35S-BBi5	35.5 ± 5.26	70.3 ± 7.98	70.6 ± 30.94
35S-KTi7	41.3 ± 5.46	76.7 ± 6.92	93.4 ± 18.94
GTP-BBi5	43.5 ± 6.42	72.3 ± 9.16	98.7 ± 22.28
GTP-KTi7	39.7 ± 15.07	75.1 ± 8.71	79.3 ± 24.04
Wild type	39.2 ± 10.78	75.4 ± 8.72	82.6 ± 23.17

Values represent mean ± standard deviation. There were no significant differences at *p* ≤ 0.05 as tested by ANOVA with SAS software v9.4.

**Table 2:** Insect population surveys in ambient insect herbivory soybean field trial containing transgenic trypsin inhibitor (TI)-overexpressing (35S-BBi5, 35S-KTi7, GTP-BBi5, GTP-KTi7) and wild type lines.

Line	Pod Feeders	Piercing–Sucking	Defoliators	Grand Total
35S-BBi5	8	87	50	**147**
35S-KTi7	11	154	42	**213**
GTP-BBi5	7	140	38	**192**
GTP-KTi7	10	141	37	**191**
Wild type	13	245	45	**304**
**Grand total**	**49**	**767**	**212**	**1047**

Each value represents the total of three population surveys. Bolded font indicates totals.

**Table 3:** Seed yield per plot of transgenic trypsin inhibitor (TI)-overexpressing (35S-BBi5, 35S-KTi7, GTP-BBi5, and GTP-KTi7) and wild-type lines in ambient deer herbivory soybean field trial.

Line	Seed Weight (g)
35S-BBi5	1112.435 ± 685.82
35S-KTi7	1276.296 ± 715.33
GTP-BBi5	1005.841 ± 642.33
GTP-KTi7	1432.218 ± 743.82
Wild type	1410.105 ± 489.88

Values represent mean ± standard deviation. There were no significant differences at *p* ≤ 0.05 as tested by ANOVA with SAS software.

**Table 4:** Defoliation of transgenic trypsin inhibitor (TI)-overexpressing (GTP-KTi7, 35S-KTi7, 35S-BBi5, GTP-BBi5) and wild-type lines in ambient deer herbivory soybean field trial.

Line	Date	Defoliated Plants (%)
GTP-KTi7	28 June	0.19 ± 0.23
	8 July	0.22 ± 0.21
35S-KTi7	28 June	0.12 ± 0.22
	8 July	0.18 ± 0.23
35S-BBi5	28 June	0.21 ± 0.22
	8 July	0.16 ± 0.25
GTP-BBi5	28 June	0.11 ± 0.15
	8 July	0.17 ± 0.22
Wild type	28 June	0.13 ± 0.17
	8 July	0.09 ± 0.16

Values represent mean ± standard deviation. There were no significant differences at *p* ≤ 0.05 as tested by ANOVA with SAS software.

**Table 5:** Controlled feeding trials with tame deer presented a choice between transgenic trypsin inhibitor (TI)-overexpressing (35S-KTi7) and wild-type (WT) soybean leaf tissue. Values represent mean ± standard deviation.

Day	Line	Consumed (g) 1st Trial	Consumed (g) 2nd Trial
1	Transgenic	135.36 ± 140.72	156.8 ± 138.62
	WT	157.48 ± 141.51	93.65 ± 137.38
2	Transgenic	121.85 ± 121.49	183.36 ± 165.87
	WT	217.32 ± 158.37	214.59 ± 163.38
3	Transgenic	107.96 ± 119.38	206.47 ± 163.97
	WT	160.39 ± 143.55	230.39 ± 154.93
4	Transgenic	207.74 ± 170.69	268.29 ± 131.23
	WT	61.28 ± 94.53	244.46 ± 170.18
5	Transgenic	94.65 ± 87.92	222.13 ± 181.17
	WT	133.95 ± 116.18	284.15 ± 146.21

Values represent mean ± standard deviation. There were no significant differences at *p* ≤ 0.05 as tested by ANOVA with SAS software.

**Table 6:** Transgenic trypsin inhibitor (TI)-overexpressing and wild-type lines used in the present study.

Line Name	Promoter	Gene Name
35S-KTi7	CaMV 35S	Kunitz trypsin inhibitor 7 (KTi7)
35S-BBi5	CaMV 35S	Bowman-Birk inhibitor 5 (BBi5)
GTP-KTi7	rbcs-SRS4	Kunitz trypsin inhibitor 7 (KTi7)
GTP-BBi5	rbcs-SRS4	Bowman–Birk inhibitor 5 (BBi5)
Wild type		

CaMV 35S: Cauliflower mosaic virus; rbcs-SRS4: green tissue promoter (GTP) ribulose-1,5-bisphosphate carboxylase small subunit gene SRS4.

## Data Availability

The original contributions presented in the study are included in the article.
